# From the harmony to the tension: Helmuth Plessner and Kurt Goldstein’s readings of Jakob von Uexküll

**DOI:** 10.1007/s40656-024-00607-9

**Published:** 2024-01-25

**Authors:** Matteo Pagan, Marco Dal Pozzolo

**Affiliations:** 1 Scuola Normale Superiore, Pisa, Italy; 2 École des Hautes Études en Sciences Sociales, Paris, France; 3 Université de Bourgogne, Dijon, France

**Keywords:** Helmuth Plessner, Kurt Goldstein, Jakob von Uexküll, Philosophical biology, Theory of the organism, Organism-environment system

## Abstract

This paper investigates the reception and discussion of Jakob von Uexküll’s biological theory by two German thinkers of his time, Helmuth Plessner and Kurt Goldstein. It demonstrates how their bio-philosophical perspectives are on the one hand indebted to Uexküll’s theory and, on the other, critical of its tendency to excessively harmonize the relationship between living beings and their environment. This original critical reading of the *Umweltlehre* is rooted in ambiguities within Uexküll’s own thought - between a dynamic conception of the organism-environment relationship and the idea of "conformity to a plan" -, , which is here examined in the second section. In the third and fourth sections we will then focus on Plessner and Goldstein respectively, demonstrating how for these two authors the harmony between organism and environment is not an original state, but only reveals itself against the background of a tension; as such, it can only be partial, unstable and always changing. The two thinkers avoid the rigid alternative between Darwin’s concept of adaptation (*Anpassung*) and Uexküll’s “fitting into” (*Einpassung*) by theorizing the ideal state of the relationship between organism and environment in terms of “adequacy” (*Adäquatheit*) and “adaptability” (*Adaptiertheit*). Between organism and environment there is neither absolute separation nor perfect harmony, but rather a gap which can never be definitively fixed.

## Introduction

The primary aim of this paper is to investigate the reception and discussion of the thought of the Baltic German zoologist Jakob Johann Baron von Uexküll (1864–1944) by two German thinkers of his time, Helmuth Plessner (1892–1985) and Kurt Goldstein (1878–1965). This will highlight some important convergences between two authors who, although sharing several theoretical insights and a solid scientific background, never explicitly confronted each other.^1^ What Plessner and Goldstein, who were forced to emigrate from Germany after the advent of the Nazi regime,^2^ have in common is not only biographical: their most significant works, respectively *Levels of Organic Life and the Human* (1928) and *The Organism* (1934), present numerous convergences with regard to the description of the organism,^3^ which can be investigated starting from their *critical reprise* of Uexküll’s theoretical biology.

As will be seen, Plessner and Goldstein often refer favourably to Uexküll’s biological theory, but at the same time emphasize some of its problematic points. Their critique differs from that of other German philosophers such as Scheler, Heidegger and Gehlen (who also refer to the Baltic zoologist), because it is not so much (or only) about the extension of the concept of environment (*Umwelt*) to the human,^4^ but primarily about the relationship between organism and environment. What Plessner and Goldstein contest is the tendency of Uexküll’s *Umweltlehre* to excessively harmonize the relationship between living beings and their environment. As is well known, according to Uexküll, nature produces all its organisms following a building-plan (*Bauplan*). Conformity to the plan (*Planmäßigkeit*) ensures perfect complementarity, complete “fitting into” (*Einpassung*), between the different organisms and their environments. Through this notion, Uexküll opposes the concept of adaptation (*Anpassung*) and the Darwinian theory of natural selection. The harmony between organisms and their environments is not produced by external modelling, but is guaranteed from the outset by a precise natural plan. This perspective, although balanced by other strands of Uexküll’s thinking, seems to leave little room for the organism’s characteristic autonomy and creativity.

On the contrary, according to Plessner and Goldstein, the organic subject does not have an a priori and already accomplished relationship with its environment, but a dynamic relationship insofar as it is *tensional* (Köchy, 2022, p. 211). In this regard, Goldstein speaks of a *Auseinandersetzung* between organism and environment,^5^ whereas for Plessner the organic subject is both *in accordance with and in opposition to* the positional field. The two thinkers thus attempt to avoid the rigid alternative between Uexküll and Darwin, between *Einpassung* and *Anpassung*, by theorizing the ideal state of the relationship between organism and environment in terms of “adequacy” (*Adäquatheit*) and “adaptability” (*Adaptiertheit*). The relationship between organism and environment is neither absolute separation nor perfect harmony, but rather presents a gap which can never be definitively fixed. This situation implies a constant confrontation between the two poles so that the relationship can be maintained. In this theoretical framework, the organism accords with its environment while retaining a margin of autonomy and unpredictability, a capacity for adaptation that the idea of pre-established harmony cannot explain. The harmony between organism and environment is not an original state, but only reveals itself against the background of a tension; as such, it can only be partial, unstable and always changing. The organism-environment system is not closed, but open, subject to constant change.

In Sect. 2, after emphasizing the originality of Plessner and Goldstein’s readings of Uexküll in the German context of the first half of the 20th century, we will show how they fit within something like a fault line, a tension that runs through the Baltic zoologist’s entire oeuvre. In Sect. 3, the complex relationship of reprise and disavowal that characterizes Plessner’s confrontation with the *Umweltlehre* will be considered, with particular emphasis on his critique of Uexküllian solipsism and its overcoming through the thematization of the organism’s leeway (*Spielraum*) and its relationship with the environment as adaptability (*Adaptiertheit*). In Sect. 4, we will focus on the theoretical operation performed by Goldstein on Uexküll. His reading consists of a resumption of empirical results and the notion of *Umwelt*, which, however, is subjected to a theoretical reinterpretation in the sign of the concepts of *Auseinandersetzung* and *Adäquatheit*. In the Sect. 5, we will emphasize the convergence of Plessner and Goldstein’s readings of Uexküll. These two prominent representatives of philosophical biology (cf. Grene, 1968) describe the relationship between organism and environment not as a stable harmony, but as a dynamic process, the result of an ineliminable tension, of a latent restlessness.

## Uexküll’s legacy and ambiguity

Jakob von Uexküll’s thought has exerted a profound influence on the scientific and philosophical landscape of the 20th century (cf. Brentari, 2015, pp. 175–231; Michelini & Köchy, 2020). His relationship with philosophy is of particular note, not only insofar as his biological theory influenced prominent philosophers, but also to the extent that it was itself strongly influenced by Kant. However, it is not so much the Kantian imprint, but rather the anti-Cartesian and, more generally, anti-mechanistic orientation of his theory that proved essential in Uexküll’s philosophical reception in Germany (cf. Uexküll, 2010a, 2010b, p. 41). In fact, the goal of German philosophical anthropology was to reunify the image of the human being, fragmented by the various human, social and natural sciences, not through the identification of a specific ontological element - such as the Cartesian *res cogitans* - but through the determination of the qualitative difference that presents human’s relationship with the sphere of life. In other words, the human differs from the relational mode that characterizes other living beings and, in particular, animals. In this framework, Uexküll’s research could make a fruitful contribution. Many philosophers used the concept of *Umwelt* precisely to describe the operational context of animal being, that is, to elaborate the theoretical background from which to determine (by opposition) the *Sonderstellung* of the human. Uexküll’s thought, which is characterized by a substantial continuity between human and animal, was thus employed against his intentions to draw a clear distinction between the environmental constraint of animals and the openness to the world of humans.

Indeed, the difference between *Umweltgebundenheit* and *Weltoffenheit* was emphasized by many of the philosophical interpretations of Uexküll’s work that were carried out in Germany in the first half of the 20th century. Similarly but not identically, Scheler, Plessner and Gehlen emphasized the closed nature of animal environments^6^, to which they opposed the human capacity to open up to the world (cf. Scheler, 2009; Plessner, 2019; Gehlen, 1988). Heidegger and Cassirer are two other examples of this of this widespread reading in Germany. The former stressed that the animal lives in a state of “captivation” (*Benommenheit*) and is therefore “poor in world” (*weltarm*), while humans are “world-forming” (*weltbildend*) (cf. Heidegger, 1995). The latter pointed out how the closure of the functional circle, when extended to the anthropological level, is inadequate and clearly distinguished the environment perceived by animals from the human symbolic world (cf. Cassirer, 1944; Cassirer, 1996). What these interpretations highlight, but do not criticize, is a problematic point in Uexküllian biological theory, namely solipsism, the idea that each animal subject lives in a “bubble” that represents its environment and contains all signs accessible to the subject. This anti-evolutionary idea appears to be marked by the metaphysical presupposition of a perfect correspondence between organism and environment that can no longer be taken as a given. Indeed, according to the enactivist philosopher Ezequiel Di Paolo, it should be radically challenged in order to ensure “a future for Jakob von Uexküll” (Di Paolo, 2020).

This point has already been raised by two of Uexküll’s contemporaries, Plessner and Goldstein, who for this reason stand out in the German landscape of the philosophical reception of *Umweltlehre*. Their reading of Uexküll’s biological theory and in particular of his description of the relationship between organism and environment cannot be described as an integral assimilation, without objection: on the contrary, it is a critical resumption. As will be seen, Goldstein’s and Plessner’s perspectives on the organism-environment relationship are indebted to the *Umwelt* theory elaborated by Uexküll in the early years of the century and then refined in the following decades. Both thinkers, however, reinterpret the Uexküllian concepts in the same mode: they both underline the gap that exists between organism and environment in order to widen the leeway of the behavioural dimension with respect to the building plan that governs the organism. This reinterpretation of the Uexküll’s doctrine is rooted in a tension inscribed in the Baltic zoologist’s own thought and it is useful here to explore its nature in more detail. On the one hand, Uexküll’s theoretical biology seems to open itself to a dynamic conception of the organism-environment relationship, leaving room for the creativity of the living individual; on the other hand, the centrality assumed by the concept of *Bauplan* and the idea of a “conformity to a plan” seem instead to fix the organism to its a priori endowment, perfectly harmonized with the specific environment. In this ambiguity, perhaps never fully resolved in Uexküll’s intellectual trajectory, resides a fruitful space for critical reflection, in which Plessner and Goldstein operate in parallel.

Regarding the first orientation within Uexküll’s work, it has been noted that the conformity between organism and environment is not to be understood in terms of a hypostasis, but rather as a dynamic relationship (Köchy, 2020, p. 55). The basic premise of the notion of *Umwelt* is, as it is commonly understood, the idea that “each and every living thing is a subject that lives in its own world, of which it is the centre” (Uexküll, 2010a, p. 45). This centrality seems essentially to be based on the organism’s ability to actively direct the specific situation to which it is subjected. A first indication of this can be found in the multiplicity of functional circles that articulate the animal’s experience, that is in the circular relationships between receptors and effectors of the organism that envelop the object. Resolutely non-mechanical, the living being’s reaction is, even in the simplest organisms, the selection of a response habit appropriate to the circumstance (cf. Uexküll, 1926, pp. 127–129; cf. Brentari, 2015, p. 103). In *A Foray into the Worlds of Animals and Humans* (1934), Uexküll reinterprets the question of different functional circles (nourishment, defence, reproduction…) starting from the concepts of mood (*Stimmung*) and effect tone (*Wirkton*), i.e., behavioural projections of the animal onto its environment. The lexicon of tone refers to a set of dispositions that can be declined by the subject according to the interpretation of the given situation (cf. Uexküll, 2010a, pp. 92–98; cf. Gens, 2014, pp. 68–70). The animal selects, assesses, interprets stimuli from the environment (cf. Esposito, 2020, p. 41). Uexküll explicitly critiques the notion of instinct, opting instead for the dimension of learning: “each new experience conditions a new attitude toward new impressions. By this means, new perception images with new effect tones are created” (Uexküll, 2010a, p. 96). Not only does the animal have at its disposal a set of behavioural orientations that shape its specific environment, but these dispositions are also at least partly susceptible to evolution and learning (cf. Brentari, 2015, p. 120). The plastic dimension of the organism, exceeding the aprioristic structure of the building plan, is a function of the complexity of the organism and in particular of the characteristics of its perceptual field. The more diverse the perceptual field the more varied the behavior, the more complex the organism and the more it has the capacity to shape its environment (cf. Uexküll & Brock, 1935; cf. Brentari, 2015, p. 90). In a number of his texts, the Baltic zoologist hints that the organism’s leeway expands in higher animals; this observation seems to imply, moreover, a greater centrality of behaviour and an openness to contingency in the animal’s experience.^7^ In the same vein, it has been noted how the concept of performance (*Leistung*) recurs repeatedly in Uexküll’s lexicon: this notion refers back to the behavioural dimension and to the priority of the effect world (*Wirkwelt*), which is never fully transcendentally determined (Gens, 2014, pp. 123–125).

Thus, if there are places in Uexküll’s work that leave room for the innovation inherent in behavioural attitudes, the concept of the building plan (*Bauplan*) and the idea of conformity to a plan (*Planmäßigkeit*) seem to orient in decidedly more aprioristic terms the relationship between organism and environment. Since Uexküll’s earliest theoretical writings, the building plan identifies the “rules of functioning embracing not only the working but also its guidance” (Uexküll, 1926, p. 271), while conformity to a plan is the knowing principle that governs the relationship between organism and environment. It is the condition of possibility of the functional circles that connote behaviour (cf. Uexküll, 1926, p. 71). Although the Baltic zoologist repeatedly criticizes the idea of finality (on the grounds that nature would not be oriented by any form of intentionality), behind the concept of conformity to a plan there seems to be concealed a form of teleology that ensures the accomplished integration between the organism and its environment (cf. Duicu, 2019, p. 91). The idea of a plan governing the animal’s behavior and its functional circles explains why Uexküll claims that “all animal subjects, from the simplest to the most complex, are inserted into their environments to the same degree of perfection” (Uexküll, 2010a, p. 50). The concept of perfection expresses the idea of an accomplished integration between the organism and its environment, by which the organism always exploits all the resources at its disposal and harmonizes without discrepancies with its outside.^8^ The building plan, Uexküll goes so far as to say, first and foremost produces the subject and with it its world-environment, as its complementary counterpart. Assuming conformity to the plan, perceptual stimuli are presented as carriers of meaning, rather than as causal agents operating on receptive organs (cf. Uexküll, 1935; cf. Uexküll, 2010b, p. 150). By placing the emphasis on *Bauplan*, the margins for a dynamic and plastic articulation between organism and environment are greatly reduced. The plan seems to configure a relatively static and predetermined relationship between the two poles, and there seems to be no room for contingency (Esposito, 2020, p. 49).^9^ Behaviour ends up depending in its structure on the plan and its declination in functional circuits, without consistent autonomy or possibility of retroacting on them.

This interpretation finds further strength in his later writings, where emerges an idea of nature that presides over all relationships, a superordinate factor that harmonizes the *Umwelten* with each other (Heredia, 2020, pp. 26–27).^10^ As he writes at the end of *A Foray into the Worlds of Animals and Humans*, “Forever unknowable behind all of the worlds it produces, the subject - Nature - conceals itself” (Uexküll, 2010a, p. 135). It is like the thing-in-itself for Kant, indeterminable; moreover, conformity to the plan itself is presented in many cases by Uexküll essentially as a knowing principle. However, if it is necessary to conceive of the *Bauplan* in order to explain the perfect “fitting into” (*Einpassung*) of organism and environment, then it is equally logical to postulate a *Naturfaktor*, a larger plan that harmoniously integrates the different plans with each other (Brentari, 2015, p. 82). Nature is interpreted by Uexküll in terms of an immense musical score that brings the plans into accord with one another, according to a predetermined pattern (cf. Uexküll, 2010b, p. 185; cf. Uexküll, 1938, pp. 64, 81–82). Following the musical explanatory model, the behavior of one animal becomes a motif within the *Umwelt* of another animal, and the relationship between them is, according to Uexküll, contrapuntal (cf. Uexküll, 2010b, p. 187). To take the example found in *A Theory of Meaning*, the spider’s web is constituted for the fly: the web integrates the archetype of the fly, that is, some of its morphological and behavioural characteristics, into its own form, like a counterpoint (cf. Uexküll, 2010b, pp. 190–194). The melody of the fly is thus, originally, also an internal motif of the melody of the spider; nature is nothing but the overall composition where the different melodies are integrated (cf. Bertolini, 2003, p. 8).

The contingent dimension of behaviour seems at this point entirely compromised or strongly marginalized. “Now I claim that the law that presides over all phenomena is the conformity to the plan (*Planmäßigkeit*). There is no phenomenon which is not somehow connected according to the plan (*planmäßig*), because every phenomenon is the outflow of a subject, and all subjects are by their nature planned (*planvoll*). Without plan there is no subject. Therefore, the conformity to the plan is the primal law, which underlies all subjects, consequently all things, and which also dominates our thoughts” (Uexküll, 1938, p. 87, translation ours), one reads in *Der unsterbliche Geist in der Natur [The Immortal Spirit of Nature]* (1938). Conformity to the plan thus assumes the status of a general law of nature conceptually close to Leibniz’s idea of predetermined harmony. Although there is no textual evidence that Uexküll knew Leibniz, many scholars have pointed out the evident convergence of some of Uexküll’s theses with Leibnizian monadology (cf. Duicu, 2019, pp. 99–100; Guidetti, 2013, p. 77; Lassen, 1939; Köchy, 2022, pp. 203–204). Indeed, the notion of *Umwelt* implies an accomplished closure on itself analogous to that of the windowless Leibnizian monad; moreover, the Baltic zoologist’s thought is perfectly in line with the doctrine of perspectivism, since in both theories every point of view on the world is always situated. As already mentioned, then, particularly in his later writings Uexküll postulates an ontologically and epistemologically superior order, guaranteeing the harmonious integration between worlds-environments (Brentari, 2020, pp. 251–252).

Although he never goes so far as to theorize a metaphysics of nature, the outcome of Uexküll’s intellectual trajectory seems to broaden and radicalize the teleological dimension already inherent in *Bauplan*’s concept. The leeway, the possible gap between organism and environment, that is, the conditions for thinking creativity on the behavioural and experiential level, is thus compromised. The latter hypothesis, which nevertheless remains a fruitful axis of Uexküll’s work, is instead re-elaborated by Plessner and Goldstein: indeed, both explore further dimensions of it by taking up some of the leading elements of the Uexküllian reflection, not without subjecting to criticism the problematic traces of Leibnizianism that have been highlighted. As has been seen, the premises of this theoretical operation are in part already present in Uexküll’s thought, more so than Plessner and Goldstein point out.

## Plessner: the organism’s leeway

In his *Selbstdarstellung* Plessner reports a meeting with Uexküll in Tartu in 1917 and states that the zoologist remembered him from his Heidelberg days, where the German philosopher studied zoology and philosophy from 1911 to 1914 under the mentoring of Hans Driesch (cf. Plessner, 1985, pp. 315–316). This encounter prompted Plessner to read the Uexküllian work carefully, by which he was considerably impressed. It is therefore not surprising that in the preface to the first edition of *Levels of Organic Life and the Human* (1928) he refers to the impulses of the “new biology” of Driesch and Uexküll, qualifying them as decisive influences on his work (cf. Plessner, 2019, p. XV). Reference to Uexküll’s thought runs through Plessner’s entire oeuvre, and it is clear that the complex relationship of contact and detachment with the Baltic zoologist plays a prominent role in his thinking (cf. Köchy, 2015; Krüger, 2020).^11^ Not surprisingly, it was Plessner himself who organized the Third German Congress of Philosophy in Bremen in 1950, dedicated to the concept of *Umwelt*. Although in the post-war period the German philosopher devoted several essays to this category (cf. Plessner, 1952; Plessner, 1983; Plessner, 2001), it is in the 1928 work that can be found his most complete account of Uexküll’s *Umweltlehre*. In *Levels of Organic Life and the Human* (1928), Plessner elaborates “an introduction to philosophical anthropology”, whose starting point is not the human being, but life. To grasp the specificity of the human within the living world to which it belongs, it is first necessary to understand the particularity of life itself. Plessner thus identifies a distinctive criterion of vitality, which he uses for the deduction of all characteristics of organic life. This criterion is not a metaphysical principle, but is based on the way in which a body dialectically realizes its boundary in relation to the surrounding environment. To indicate this fundamental property of life, Plessner elaborates the category of “positionality” (cf. Plessner, 2019, p. 121). This concept is the core of Plessner’s philosophical biology and the basis of the argumentative structure of *Levels of Organic Life*. What distinguishes the three levels of the organic life (plant, animal and human being) is precisely their positionality, which varies according to the form of the living body.

Within this framework, the Uexküllian biological theory is used predominantly in a positive way to define the living being as a teleologically organized unit (cf. Plessner, 2019, p. 158) and, above all, to describe the particular form of life that is the animal. According to Plessner, “the sensory- motor schema, the “function- circle”, as Uexküll calls it, is the condition of the possibility of the reality of the closed form, the organizational idea of the animal. This idea allows us to understand all the essential characteristics of animal life in their unity” (Plessner, 2019, p. 212). In the sixth chapter, devoted to the sphere of animality, references to the second edition of *Umwelt und Innenwelt der Tiere* (1921) are indeed numerous and precise. Plessner’s debt is reflected in the incorporation of specific elements of the *Umweltlehre* in the description of the animal and in the resumption of the Uexküllian terminology, albeit with some significant modifications. Specifically, Plessner takes up the idea of the plurality of environments associated with different animal species and the distinction between the “world of noticing” (*Merkwelt*) and the “world of effecting” (*Wirkwelt*) (which he reformulates as *Merksphäre* and *Wirkungssphäre*^12^). He also reaffirms Uexküll’s principle that the presence of an organizing center in higher animals leads to an increase in the extent of the perceptual sphere and takes up his distinction between the “pure signal field” of lower animals and the more stable and structured environment of higher animals, which thus gain greater freedom and at the same time greater insecurity (cf. Plessner, 2019, pp. 227–235).

In general, the Uexküllian approach seems particularly suitable to describe the situation of the animal with a decentralized organization (cf. Rasini, 2008, p. 154). Indeed, the appearance of a central organ creates new possibilities for *breaking* the association between stimulus and response. The nervous system “posits, from case to case, the *interruption, inhibition, interval* (between stimuli and response), *which in positional terms is* the being of a self in the central position – that is, its being “against something in the surrounding field” or its intuition of something” (Plessner, 2019, p. 242). Therefore, the sphere of consciousness represents “the hiatus, the void, the internal chasm through which the response follows upon the stimulus” (Plessner, 2019, p. 227). However, it is important to point out that for Plessner the functional circle is not configured as a closed monad-like order even at the level of lower animals (see Köchy, 2022, p. 209). The reaction of animals to the environment, schematized in Uexküll’s functional circle, is effective only if there is a break between perception and action. Otherwise, it would make no sense to notice something in order to act on something. The insertion of the organism into the environment “is not an absolutely rigid bond […], but rather a bond within certain boundaries that provide the living being with leeway [*Spielraum*]” (Plessner, 2019, p. 228). Even for the decentralized organization the connection between perception and action cannot therefore be thought of as an immediate relationship. On the contrary, there is a “strange relationship of indirect directness, of mediated immediacy between the organism and the world, already expressed in the essence of the closed form and profoundly grounded in life’s structure of being” (Plessner, 2019, p. 241).^13^

As has been noted, the recognition of ruptures, of emptiness, of unharmonized tensions, distinguishes Plessner’s description of the relationship between organism and environment from that of Uexküll and, moreover, implies a different consideration of animal psychology (cf. Schmieg, 2017; Köchy, 2022, pp. 203–211). Indeed, the German philosopher does not share the Baltic zoologist’s hostility to animal psychology. While giving Uexküll credit for critiquing naive anthropomorphism and thus enabling modern biology to elaborate not a “crypto-psychology”, but a “a phenomenology of living behaviour” (Plessner, 2019, p. 58), Plessner emphasizes how his limitation of inquiry to the empirical realm eliminates any possibility of an authentic understanding of the life project. This is based on “vital categories”, whose systematic grounding constitutes “the task of a philosophical biology as the science of the essential laws of life, as well as of the foundational discipline of a possible animal psychology” (Plessner, 2019, p. 61). This discipline must avoid both the anthropomorphic descriptions of the animal’s soul and Uexküll’s maximum program, which would reduce questions of animal psychology to problems of stimulus physiology. However, Uexküll himself does not pursue his program in this sense, and, according to Plessner, was “the first to declare the relationship between the organism and its environment to be the domain of an animal psychology (biology, life plan research) brought to reason. This young science has gone beyond him, however, in the sense that (unlike the “Kantian” Uexküll) it strives to understand this relationship in its vitality and intelligibility” (Plessner, 2019, p. 63).^14^

This mention by Plessner of the Kantian matrix of the *Umweltlehre* allows us to highlight what in the philosopher’s eyes is the main problem: “Uexküll is wrong when he writes, “The environment as reflected in the counterworld of the animal is always a part of the animal itself, constructed by its organization, and processed into an indissoluble whole with the animal itself. […] The environment is properly understood only as a projection of its counterworld”. It is true that nature does not force animals to adapt, but neither do animals form nature according to their needs. That would amount to zoological idealism” (Plessner, 2019, p. 240; he quotes Uexküll, 1985, pp. 234–235). This objection is also made in the third section of the fifth chapter, in which Plessner refers to Uexküll’s theory to compensate for the one-sidedness of the Darwinian theory of evolution. The latter conceives of the relationship between organism and environment merely as a struggle for existence in which the organism plays an essentially passive role toward the environment. However, the Uexküllian position appears to Plessner equally one-sided, only in the opposite direction (cf. Krüger, 2020, pp. 89–90). If for Darwin “the organism in its positional field would find itself as if in a zone of total alienness, unpredictability, and independence: isolated and at the same time abandoned to an absolute transcendence” (Plessner, 2019, p. 189), for Uexküll “the positional field would actually belong to the organism, just as Kant’s forms of intuition of space and time belong to the subject and only the subject. The organism would then move in its positional field like the monad in the world, like a solipsist; in a surrounding field, but not in the actual world independent of it: in absolute immanence” (Plessner, 2019, p. 188). The problem is that both theories fail to see that life is essentially adaptedness (*Angepaßtheit*) *and* adaptation (*Anpassung*) at the same time (Plessner, 2019, p. 192). Darwin and Lamarck consider the relation of the organism to the environment solely from the point of view of adaptation and thus ignore the primary agreement between the living being and its vital sphere, whereas comparative physiology has considered this aspect as well, “not, it is true, without tending toward the other extreme of espousing the absolute adaptedness of life systems, giving way, as it were (as in the work of Uexküll), to a biological monadology” (Plessner, 2019, p. 192).

Plessner polemically brings Uexküll closer to Leibniz’s monadology also in his lectures in Cologne in the winter semester of 1930–1931. After recalling that according to Uexküll’s biological theory the organism is first and foremost adapted (*angepaßt*) and “fitted into” (*eingepaßt*) a given environment, he writes: “Uexküll has gone very far in this. He has once again brought the idea of adaptedness to the fore. […] He almost exaggerates this static thought of being fitted in, of being completely nestled in, that there is hardly any collision between the individual types of organization” (Plessner, 2002, p. 114). The extreme consequence of this approach is “a kind of biological monad theory (*Monadenlehre*). […] The organization conditions a perfect closedness (*Abgeschlossenheit*) of the life circle. The organism is closed (*abgeschlossen*) and enclosed (*eingeschlossen*). The organism goes around in the world like a snail with a snail shell” (Plessner, 2002, pp. 114–115, translation ours). In other words, the organism fits into its environment like molten metal into its form. In this way, however, the organism’s exposure to contingency is lost and, with it, “the dynamic invariable (Buytendijk), the range of arbitrariness, and the unpredictability of the organism” (Plessner, 2019, p. 188) - in short, its autonomy.

For Plessner there is no doubt that there is a primary adaptedness between the living body and its positional field, in which it plays an active and not merely passive role, but to avoid the risk of solipsism he decides to call it the “opposing field” (*Gegenfeld*).^15^ Here the disagreement with Uexküll comes to the fore: the subject’s counterpart is not its mere projection. This idea can only lead to an idealistic position whereby the animal subject would integrally constitute its environment. In opposition to Uexküll’s “zoological idealism”, Plessner claims the ontological independence, the value of reality, of the animal environment; this is why he speaks of *Gegenfeld*. This notion allows the German philosopher to highlight the presence of a *gap* between organism and environment. Their relationship must be understood on the basis of the essence of positionality, whereby the organic body is “both into the body that it is and beyond it”. Consequently, the living being is *in accordance with and in opposition to* the positional field; it is placed *with it and against it*. Therefore - Plessner continues - , “the positional field or milieu is, in its essence, the scene of struggle and the sphere of protection”, in relation to which the organism is the “excentric central point” (Plessner, 2019, pp. 192, 188). In this way, the positional field is neither entirely separate from the organism nor a mere reflection of its organization: “the hiatus between the organism and its setting is not destroyed, but rather bridged” (Plessner, 2019, p. 189) only momentarily. Consequently, adaptedness and adaptation could be separated only at a later stage: “there is adaptation (*Anpassung*), like fitting in (*Eingepaßtheit*), that is, an organism can succeed in adapting to a change in the environment, or it can fail. But this success or failure already occurs in a predetermined sphere, in a sphere that shows it the way from the beginning” (Plessner, 2002, p. 118, translation ours).

What guarantees an effective synthesis of these two essential properties is, according to Plessner, the union in one and the same property, in the “capacity for adaptation (*Adaptationsfähigkeit*), that is, adaptability (*Adaptiertheit*)” (Plessner, 2019, p. 189, translation modified); this concept, evidently alternative to the notions of adaptation (*Anpassung*) and “fitting into” (*Einpassung*), indicates that “within certain limits, the organism *harmonizes* in substance and form with the medium *without thereby entering into an absolute bond*” (Plessner, 2019, p. 190) which would suppress its dynamic dimension. Within this theoretical framework it is then again possible to attribute to the living being a form of autonomy: “the organism has to fit into the medium and at the same time have enough leeway in it to not only exist within the fixed forms of harmony but also to survive dangers with them” (Plessner, 2019, p. 190). Though the adaptation of the organic individual to the positional field is given in advance in form, it is not guaranteed in content; “the organism remains endangered, regardless of how secure it is […] in peace and at war, in life and in death. That is why life means to be in danger, why existence means risk” (Plessner, 2019, p. 192). Every confrontation of the organism with the surrounding field is then a risky vital act.

Ultimately, Plessner describes the relationship between organism and environment as having a latent antagonistic character. This tension is insurmountable; only an *unstable* balance is possible between the two poles. Between organism and environment there is neither perfect harmony nor total separation, but rather an oppositionally structured relationship: “life is being that is set off against the sphere of its non-being, that relates to it as to its opposite and contrary. […] This allows the boundaries of the two zones of being to open up to mutual influence without the boundaries being destroyed. Autonomy does not turn into heteronomy but is preserved by virtue of heteronomy” (Plessner, 2019, p. 196). This oppositional relation guarantees the essential openness of the organism-environment system, which cannot but change permanently: “Adaptedness is one of the prerequisites of organic life in the world, but it cannot go so far as to rob the organism from the beginning of every possibility of improving or modifying itself. […] just as the body possesses its own boundaries by at the same time being out beyond them and opening them, the closed system must too be an open system, allowing for and demanding constant correction” (Plessner, 2019, p. 191). In other words, the relationship between organism and environment is not harmoniously fixed, but always under tension and, as a result, subject to constant change.

## Goldstein: the becoming at the heart of the *Umwelt*

Uexküll’s influence on Goldstein’s thought is direct and relevant, as is evident from the significant references found in *The Organism* (1934). The concept of *Umwelt*, central to both Goldstein’s theory of the organism and theory of the pathological, is explicitly drawn from Uexküll’s work: “accurate investigations have shown that the individual organism is always fitted into a very specific environment, and that its existence, in spite of all variability, hinges ultimately on an environment that is adequate for it. Uexküll’s research is basic to this point and is so generally valid that it no longer meets with much opposition. In cases of brains injuries, our experience has everywhere shown the equivalent results” (Goldstein, 1995, p. 84). Even if there are few citations overall, Goldstein was familiar with both Uexküll’s empirical research in the early 20th century and some of his later theoretical works, certainly *Theoretical Biology* (cf. Golstein, 1995, pp. 73, 84, 88–90, 106, 343). The central idea that Goldstein inherits from Uexküll is the description of the environment as a space centred on and organized by the organism: “An environment always presupposes a given organism. […] Environment first arises from the world only when there is an ordered organism” (Goldstein, 1995, p. 85). A condition for thinking the environment, understood as *Umwelt*, is the organism as a perspective center. From this realization also follows the reprise of the distinction between the environment organized by the organism (*Umwelt*) and the purely objective physical-geographical environment (*Umgebung*): “we must make a clear distinction between the surrounding world, in which the organism is located, and the milieu that represents only a part of the world - that part that is adequate to it, that is, that allows for the described relationship between the organism and its environment. Each organism has its *milieu*, as Jakob von Uexküll has emphasized” (Goldstein, 1995, pp. 105–106). Uexküll’s *Umweltlehre* represents a point of no return, a conceptual turning point from which any theory of the living must follow.

But Uexküll’s work is also taken up by Goldstein in its empirical dimension, particularly with regard to the study of nerve excitation. As is well known, Goldstein’s research aims first and foremost at an empirical and epistemological critique of the concept of reflex. This concept is considered by the German neurologist to be an abstraction with respect to the complexity of the organism’s response, or a phenomenon typical of pathological states (cf. Goldstein, 1995, p. 74). Goldstein refutes the explanatory centrality that the concept of reflex and its derivations (such as that of the reflex arc) have assumed.^16^ He proposes instead a *Gestalt* model of the organism, according to which the response to the local stimulus is comprehensible only against the background of the organism’s wholeness (cf. Goldstein, 1995, pp. 186, 298–300). As much as Uexküll’s empirical research alone does not allow us to overcome the reflex arc model,^17^ it provides valuable material for interpreting the behaviour of Goldstein’s patients with brain injuries. For example, what Goldstein calls “the theorem of Uexküll” (Goldstein, 1995, p. 89) that is, the conception of excitation as a transferable fluid, makes it possible to explain why if excitation is prevented from propagating in one area (due to impairment or artificial impediment) it spreads to another area of the organism (cf. Goldstein, 1995, pp. 89–90). More generally, the research on the nervous system and muscle contraction carried out by Uexküll and his close collaborators (such as Albrecht Bethe) is a relevant source of Goldstein’s thought (cf. Goldstein, 1995, pp. 73, 87–93).^18^

The Baltic zoologist is thus an important reference for Goldstein and yet, without disavowing his fruitfulness, the German neurologist makes two mutually articulated objections to Uexküll’s position. The first relates to the idea that the organism is isolated “into a segregated (‘insulated’) part of the world.” He writes: “but actually the situation is not like this. Each organism lives in a world that by no means contains only such stimuli as are adequate for it. It lives not merely in its ‘own environment’ (*milieu*) but in a world in which all possible sorts of stimuli are present and act on it. The organism must cope with this ‘quasi-negative’ environment (*negativ Umwelt*)” (Goldstein, 1995, p. 85). In this passage Goldstein evidently targets the monadological temptation that innervates the *Umweltlehre*, challenging the idea of a harmonious complementarity between organism and environment (cf. Gens, 2014, p. 40). Indeed, the organism’s environment is continually shaken by its outside, in a permanent state of tension produced by stimuli that are not integrated a priori into the organism’s structure.^19^

This state of tension is reinforced by a second point raised by Goldstein, which concerns the internal dynamism of the very notion of *Umwelt*: “the environment of an organism is by no means something definite and static but is continuously forming commensurably with the development of the organism and its activity. One could say that the environment emerges from the world through the being or actualization of the organism” (Goldstein, 1995, p. 85). According to Goldstein, the organism-environment relationship cannot be static, rigidly articulated by a predetermined plan, but changes with the organism itself. It restructures itself according to the behaviour of the organism (cf. Gens, 2014, pp. 19–20; Ostachuk, 2020, p. 161). The intrinsic dynamism of this relationship is precisely summarized by the notion of *Auseinandersetzung* that runs through the pages of *The Organism*: this concept expresses the idea of a confrontation between organism and environment, as in an open dialectic between two mutually influencing poles (cf. Goldstein, 1995, pp. 10–12).^20^ Even if the concept of *Auseinandersetzung* does not directly imply conflict between the two poles, it does, however, entail a permanent gap and tension. Goldstein, in other words, emphasizes the becoming of the *Umwelt*, its continuous genesis, and not only its structural dimension (cf. Heredia, 2020, p. 31).

These two critiques, concerning the monadological closure of the *Umwelt* and its static nature, are convergent and closely integrated. The dynamism characterizing the dialectic between organism and environment is on the one hand an effect of the changing behaviour of the organism; but on the other hand is also a consequence of that “quasi-negative environment” that exposes the *Umwelt* to the unpredictability of non-predetermined external stimuli, compromising the harmonious agreement between the two poles (cf. Goldstein, 1995, pp. 101–102). In this framework, the sphere of behaviour acquires a central space, particularly in the thematization of preferred behaviour (cf. Grene, 1968, p. 227). Instead of entrusting the integration of organism and environment to the *Bauplan*, Goldstein conceptualizes patterns of behaviour that are inscribed in the *Umwelt* as functional, safe and adequate for the organism. These preferred attitudes are endowed with relative stability, but they change according to the orientation of the organism and can restructure themselves when subjected to new stimuli in a lasting way (cf. Goldstein, 1995, pp. 281, 287). Rather than transcendental structures, therefore, they are pragmatic arrangements that preserve a margin for behavioural innovation by the individual – especially in higher animals, whose range of preferential postures is wide (cf. Ostachuk, 2020, p. 161).

Goldstein’s ambivalent theoretical gesture with respect to Uexküll’s theory (acceptance and, at the same time, substantial reform) is replicated in regard to the concept of “fitting into” (*Einpassung*). If the condition of possibility of the relationship between organism and environment is the perfect conformity to a plan, the concept of *Einpassung* ends up identifying merely an agreement that is present from the beginning. It identifies an adaptedness without progressive refinement (cf. Uexküll, 1926, pp. 314–315; Gens, 2021, pp. 67–68). The primary adaptedness between organism and environment is incompatible with the Darwinian idea of adaptation (*Anpassung*) as the progressive development of characters adapted to the environment, and Uexküll and Goldstein converge on this point in a critical vein. The concept of (*Anpassung*), interpreted in strictly Darwinian terms, presupposes a single environment to which the organism should adapt, but this idea is evidently incompatible with the plurality of the *Umwelten* (cf. Ostachuk, 2020).^21^ On the other hand, for the reasons already given, Goldstein could not understand the relationship between organism and environment in an essentially static and predetermined way and must therefore refer to a third concept to name the model of adaptation suitable for his theory. This third way is represented by the notion of “adequacy” (*Adäquatheit*), which expresses the organism’s ability to relate orderly, appropriately, to its environment. “An organism that actualizes its essential peculiarities, or - what really means the same thing - meets its adequate milieu and the tasks arising from it, is “normal.” Since this realization occurs in a specific milieu in an ordered behavioral way, one may denote ordered behaviour under this condition as normal behavior” (Goldstein, 1995, p. 325). An adequate environment identifies a temporary, fragile and always risk-exposed state of adaptation that allows the organism to express its behavioural attitudes (cf. Goldstein, 1995, pp. 104–106). The adequate environment corresponds to ordered behaviour, that is, a behavioural pattern that conforms to the demands of the environment in a given situation (cf. Goldstein, 1995, pp. 290–291; Goldstein, 1963, pp. 88–89). Ordered behaviour can, however, turn into a catastrophic reaction if a too wide a gap is created between environmental stimuli and the organism’s ability to cope with them (cf. Goldstein, 1995, p. 105). Thus, not only can adaptation between organism and environment never be said to be complete, as it is structurally exposed to instability, but there can also be sudden reconfigurations of the relationship, like quantum leaps from one state to another.

Evidence of this conception is the transition from the healthy state (ordered behaviour) to the pathological state (catastrophic reaction), a form of alteration of the *Umwelt* that is of particular interest to Goldstein. “In its tendency to maintain optimal performance and to attain new ordered functioning, the diseased organism either adapts itself to a less relevant defect by yielding to it, or adjusts itself to a stronger defect by reorganizing the impaired performance at the expense of others (shift). In either case, the new order necessitates a shrinkage or diminution of performance potentialities (essential nature) and of milieu” (Goldstein, 1995, p. 344). Health and disease are two norms that articulate living and specific environment in qualitatively different ways (cf. Goldstein, 1995, p. 105; Goldstein, 1971, p. 8). The pathological state is in fact a reorganization of the organism-environment relationship, in the sense of its restriction, that is, a reduction of the individual’s leeway. Healing represents the recovery of ordered behaviour and adequate environment, but it is a new norm of living, not referable to the previous ones (cf. Goldstein, 1995, p. 350; Ostachuk, 2020, p. 162). The experience of the pathological highlights how the *Umwelt*, far from static and predetermined, can modify itself qualitatively and then restructure itself again according to variable geometries.

This example confirms the idea of a dynamic confrontation between organism and environment. The choice of the term *Adäquatheit*, which is not without ambiguity, should not lead one to think that the organism naturally tends to seek a state of equilibrium with its environment, that is, that it is moved essentially by a principle of preservation of the ordered state (cf. Ostachuk, 2020, p. 169). According to Goldstein, the fundamental drive of the living is self-realization, the development of the organism’s creative potential, and not conservation (which characterizes pathogenic forms of adaptation instead). The drive for self-realization is affirmed by exposing the individual to uncertainty, trauma and resistance (cf. Goldstein, 1963, pp. 111–112). In other words, perfect conformity between organism and environment is made structurally impossible by the very tension of the living being to self-affirmation. This pushes the organism to overcome the mere spirit of preservation, while also exposing itself to the risk of falling back into a catastrophic situation and the resulting anxiety. The organism nurtures its own capacity for innovation, its own creativity, at the price of a permanent state of tension, of a perpetual exposure to the restlessness of contingency.

## Conclusion

In our concluding remarks, we would like to emphasize that the theoretical operation performed by Plessner and Goldstein on Uexküll is surprisingly synchronical, considering the very limited influence the two thinkers exert on each other. They both share a solid background in the life sciences (in particular biology and medicine) and an original theoretical work on the theory of the organism, which orients their reading of the *Umweltlehre* and distinguishes it from other philosophical receptions in the German context. Indeed, Plessner and Goldstein’s approach to the problem of the organism draws equally on philosophy and scientific practice. The former is a philosopher, a student of the biologist Hans Driesch, who had undertaken, but not completed, a doctorate in zoology and collaborated on several occasions with his friend and colleague Buytendijk, in both experimental and theoretical research projects.^22^ The second, on the other hand, is a neurologist whose thought assumes an intertwining of clinical research and philosophy. Moreover, the results of his experiments have been an indispensable reference for philosophers such as his cousin Cassirer and, in France, Canguilhem and Merleau-Ponty. Most importantly - in Hans Jonas’s words - “Kurt Goldstein is a philosophical scientist because he is a true scientist. With no other intention than that of advancing the understanding of his particular subject, his work assumes philosophical significance, and his statements have the style of philosophic statements” (Jonas, 1965, p. 352) It is therefore no coincidence that the approaches of Plessner and Goldstein show significant affinities with the research of the Dutch physiologist and psychologist Frederik Buytendijk and the Swiss biologist and zoologist Adolf Portmann. The theoretical program of these four authors (and Erwin Straus), at the intersection of the natural sciences and philosophy, has been called philosophical biology (cf. Grene, 1968) and is closely related to Uexküllian thought. These authors explicitly recognize the importance of Uexküll’s theoretical biology for the study of animal behaviour and see in the Baltic zoologist a forerunner in the research of a third way between the anthropomorphism of animal psychology and the extreme reductionism of the behaviourist approach.

Indeed, as noted above, Plessner and Goldstein place the Uexküllian theory at the center of their own oeuvre. However, they simultaneously operate a critique and reform of the concept of *Umwelt* along the same theoretical lines. Both replace the Uexküllian view of harmonious relations in nature with an evolutionary view of the natural world, but, in contrast to Darwinian evolutionary theory, understand the autonomy of living beings as a dynamic tension with the environment rather than as an open conflict with it.^23^ The convergence of this reinterpretation is permitted by an internal tension in Uexküll’s thought, in which Plessner and Goldstein theoretically discover a laboratory of conceptual creation guided by the same opposition to the monadological temptation. However, this converging critical gesture remains inscribed in the track opened by Uexküll, whose legacy represents an unavoidable new scientific and philosophical foundation for an entire generation of thinkers. Indeed, the notion of *Umwelt* proposed by Uexküll is a significant turning point on the concepts of “*milieu*” and “environment” that circulated in Western Europe in the late-19th and early-20th centuries. These concepts were strongly influenced by French positivism, whose perspective tend to identify the environment with the set of external circumstances that constitute the individual.^24^ Within this context Uexküll, over the years 1909 and 1910, proposed the concept of *Umwelt* in explicit polemic with French positivism and in particular with the concept of *milieu* provided by Hippolyte Taine (cf. Feuerhan, 2009; Feuerhan, 2017). As evident from what has been discussed above, Uexküll’s proposal represents a theoretical alternative to the positivist reading and, for this reason, was able of opening up new research perspectives. Goldstein and Plessner, like others at the time and later thinkers, owe to Uexküll the opening of a theoretical space from which it was possible to reinterpret the empirical results of the sciences contemporary to them. The conceptual operation carried out in parallel by the two authors is thus to be understood as a reform of the *Umweltlehre* and its implications, which confirms the fruitfulness of this notion, rather than rejecting it. It is by prolonging some of the insights in Uexküll’s thought that, after all, the fundamental elements of Goldstein and Plessner’s positions are to be found. Ultimately, the two authors decouple the heuristic and operational significance of the notion of *Umwelt* from the inclination toward idealism and pre-established harmony that runs through Uexküll’s work.

Indeed, Goldstein and Plessner independently propose a reform of the concept articulated on two closely related critiques. As we have demonstrated, the harmonious complementarity between organism and environment conceived by Uexküll is shaken by an internal tension that inserts tragic notes into the musical score that is the living. Even if the *Umwelt* remains the space organized by the organism and adapted to its needs, its morphology is more fragile and susceptible to reconfiguration than it was for the Baltic zoologist. The concepts of *Adaptiertheit* and *Adäquatheit* complement Uexküll’s critique of the *Anpassung* (through the notion of *Einpassung*), in that it assumes the existence of only one universal environment. However, compared to the Uexküllian concept, they both emphasize the precarious and temporary state of the balance between organism and environment. The consequence is a restlessness that inhabits the living being, but also a greater openness in terms of behavioural creativity. This fundamental objection to Uexküll, guilty of overestimating the perfection of the organism-environment system, actually has as its premise another critique, which focuses on the reduction of the *Umwelt* to a projection of the subject. Respectively with the concepts of the opposing field (*Gegenfeld*) and the negative environment (*negativ Umwelt*) Plessner and Goldstein emphasize an excess of the environment over the organism’s perceptual-operational complex. In other words, the *Umwelt* is the emergent result of an open dialectic between the organism and a larger environment endowed with relative ontological independence, rather than a component of the monad-organism^25^. In this way, the *Umwelt* becomes the result of a pragmatic negotiation with the “outside” of the individual and is not reducible to a purely constituted structure in terms of an idealistic-transcendental perspective.

This second critique is logically connected to the first because subtracting the *Umwelt* from a purely transcendental interpretation opens up the possibility of a tension between organism and environment. What puts the relationship in tension is the existence of a counterpart that is not reducible to the organism, which therefore does not fully collimate with its a priori endowment. It is also and especially because the *Umwelt* is embedded in a larger “outside” that it is exposed to variation and consequently can reconfigure itself. The behaviour of the organism changes in response to the variations in the opposing field or the negative environment and in some cases imposes modifications on it. The relative independence of the environment makes possible a real organism-environment dialectic that from an idealistic point of view would instead be nonexistent or already resolved in the subjective pole. At the point of articulation of this double critique Plessner and Goldstein’s perspectives manifestly converge, reforming the concept in the same direction. Probably without ever having become aware of it, the two thinkers walk the same path, heirs and betrayers of Uexküll’s thought in the same measure. The result of this critical reprise is functional for the two authors to reflect on different problems, within different research programs, but undoubtedly grounded on the same concept of *Umwelt*.

## Footnotes

1 Plessner refers only to the studies conducted by Goldstein and Gelb on aphasia and apraxia, while Goldstein merely makes quick mention of Plessner in an essay on smiling (cf. Plessner, 2019, pp. 262, 336; Goldstein, 1957).

2 Both Plessner and Goldstein emigrated to the Netherlands, respectively to Groningen and Amsterdam. Plessner did not return to Germany until 1951, while Goldstein moved to the United States in 1934 and remained there until his death in 1965. In her detailed reconstruction of Plessner’s biography, Dietze points out that the German philosopher, while holding the Theodor Heuss Chair at the New School for Social Research in 1962, was introduced to emigrant circles in New York; in these circles Plessner met Goldstein (cf. Dietze, 2006, p. 512).

3 It is surprising that Plessner and Goldstein themselves, who also shared a strong friendship with Frederik Buytendijk, did not notice this. Probably because of the limited cross-references between them, a systematic comparison of their theories of the organism has not been worked out so far. However, some important points of agreement between the two authors have been highlighted by Ebke, Köchy, and indirectly by Grene (cf. Ebke, 2012, pp. 346–356; Köchy, 2022, p. 211; Grene, 1968, pp. 257–282).

4 This is a veritable *topos* of German philosophical anthropology, which also runs through all of Plessner’s thought since *Levels of Organic Life and the Human*. Goldstein does not make this critique of Uexküll, but he does not dismiss the anthropological difference (cf. Goldstein, 1995, pp. 353–375).

5 This concept, which in German is usually used to denote confrontation, debate and discussion (even polemical), has been translated in the English Edition of *The Organism* (published in 1939) as “coming to terms”.

6 As will be seen, for Plessner the animal environment is closed only in comparison with the human openness to the world.

7 Clearly moving in this direction is Merleau-Ponty’s interpretation of Uexküll: according to the French philosopher, the Baltic zoologist is a forerunner of the notion of behavior and a theorist of the “living plan”, rather than of transcendent finality. Cf. Merleau-Ponty, 2003, pp. 167–178.

8 There is reorganization of constructive plans and functional circuits only when a new species emerges; otherwise the animal is completely integrated with the environment, ontogeny corresponding rather to a process of increased specialization of functions. Cf. Guidetti, 2015, pp. XLVIII–XLIX; Uexküll, 1926, pp. 163–166.

9 By openness to contingency we do not refer here simply to limited margin of manoeuvre through which the animal declines its species endowment; we refer to an inventive capacity with respect to the structure of behaviour, which is not transcendentally constrained.

10 Indeed, reference to this idea is already made in *Theoretical Biology*, albeit not extensively. Cf. Uexküll, 1926, p. 129.

11 Plessner refers to the Uexküllian biological theory also in two articles written with Frederik Buytendijk (cf. Plessner & Buytendijk, 1982; Plessner & Buytendijk, 1983) in order to criticize the concept of reflex and mechanism in general. Particularly in the essay entitled *Die physiologische Erklärung des Verhaltens: eine Kritik an der Theorie Pawlows* [The physiological explanation of behaviour: a critique of Pavlov’s theory] (1935) Plessner and Buytendijk develop a critique of the explanation of behaviour in terms of reflexes (*erklärende Physiologie*) and formulate a new comprehensive theory of behaviour (*verstehende Physiologie*). While the former reduces behaviour to a mechanical reaction, the latter considers the environmental intentionality (*Umweltintentionalität*) of behaviour and, in so doing, makes its meaning comprehensible.

12 This is because Plessner uses the expression *Wirkungswelt* to designate the “world of effecting” in Uexküll’s functional cycle (see Plessner, 2019, p. 230).

13 The Hegelian concept of “mediated immediacy” could be considered the key to Plessner’s philosophy. It is no accident that it is one of the three basic anthropological laws set forth at the end of *Levels of Organic Life and the Human* (see Plessner, 2019, pp. 287–316).

14 Plessner refers here to the important researches of Köhler, Katz, and Buytendijk; in the article written in 1925 with the latter, there is an explicit critique of Uexküllian biology, whose “leveling according to the world of noticing – inner world – world of effecting [*Merkwelt-Innenwelt-Wirkungswelt*] scheme will never do justice to the *change* of meaning of the outer world, i.e. to the variability of the inner and outer form and thus of the stimulus values” (Plessner & Buytendijk, 1982, p. 74, translation ours).

15 As will be seen, this notion has structural analogies with the “quasi-negative environment” mentioned by Goldstein.

16 This is a relevant convergence with Plessner and Buytendijk: see note 11. Cf. Grene, 1968, p. 241.

17 Moreover, this was not the goal of Uexküll’s physiological research, especially the work dating back to the early years of the century. On Uexküll’s intellectual path and its discontinuities see Heredia (2020).

18 Köchy has remarked that there is also a convergence between Uexküll’s research on antagonistic muscles and the argumentation developed in *The Organism* that focuses precisely on physiological antagonisms (although Goldstein’s direct reference in this regard is Sherrington). Cf. Köchy, 2020, pp. 53–54.

19 The concept of *negativ Umwelt* mentioned by Goldstein could be associated with Plessner’s *Gegenfeld*. Both concepts express the irreducibility of the *Umwelt* to the organism’s perceptual-operational projection alone, connoting the environment in a more realistic sense than in Uexküll.

20 Georges Canguilhem in “The living and its milieu” (1952) insists on this notion, revealing his debt to Goldstein’s reading of the *Umweltlehre*. Cf. Canguilhem, 2008, pp. 113–114.

21 It should be pointed out here that the relationship between Uexküll and the evolution is more complex than has sometimes been reconstructed, since Uexküll’s antidarwinism does not imply a denial of evolutionism. Uexküll explicitly challenges the idea of evolution at the level of the individual, but he does not exclude the evolution of the species by leaps, through restructuring of the building plan. Cf. Uexküll, 1926, pp. 263–269; Guidetti, 2015, p. XLIX; Gens, 2021, p. 69.

22 Also Goldstein was linked to Buytendijk by a long-standing friendship; in *The Organism* he states his agreement with Buytendijk’s description of animal behaviour. Cf. Goldstein, 1995, p. 355.

23 As should be clear from the foregoing, the concept of autonomy for Goldstein and Plessner has a partially different meaning than that of Uexküll, from which the different objections to Darwin derive. Although in Uexküll there’s room for the behavioral dimension, autonomy is declined by the Estonian zoologist in a transcendental sense in relation to the *Bauplan*; in Plessner and Goldstein autonomy is essentially associated with the unpredictability of the organism and the environment. There is in this regard a strong convergence with the research of Adolf Portmann. Cf. Portmann, 1990, p. 460. Cf. Jaroš & Brentari, 2022, p. 7. On Portmann’s critique of Uexküll cf. Portmann, 1956, pp. 13–17.

24 The positions of positivists, for example those of Auguste Comte and Hippolyte Taine, should be distinguished. On these aspects cf. Braunstein, 1997; Taylan, 2018. On the concept of environment see Pearce (2010).

25 In this regard there is a greater possibility of reconciliation with the extended evolutionary synthesis (Laland, Uller, Feldman, Sterelny, Müller, Moczek, Jablonka, & Odling-Smee, 2015) than starting from the Uexküllian *Umwelt*. In particular, a remarkable convergence can be observed between Plessner and Goldstein and the niche construction theory (Odling-Smee et al., 2003), which considers the organisms as co-directing their own evolution. This perspective was originally introduced into evolutionary biology through the pioneering work of Lewontin, who in a book not coincidentally entitled *The Dialectical Biologist* (written with Levins) emphasized the “complex dialectical relationship of the elements in the triad of gene, environment, and organism. […] The organism is, in part, made by the interaction of the genes and the environment, but the organism makes its environment and so again participates in its own construction” (Levins & Lewontin, 1985, p. 105). Similarly, as we have seen, for Plessner and Goldstein the organism-environment system is not a simple adjustment of the former to the latter (Darwin’s *Anpassung*), nor of the latter to the former (Uexküll’s *Einpassung*), but a reciprocal, mutually formative relationship.
